# Surfeit folic acid, protein, and exercise modify oncogenic inflammatory biomarkers and fecal microbiota

**DOI:** 10.3389/fnut.2022.1060212

**Published:** 2023-01-19

**Authors:** Rebecca M. Tuska, Sophia M. Helm, C. Foster Graf, Courtney James, Gabriel Kong, Leah T. Stiemsma, David B. Green, Susan Edgar Helm

**Affiliations:** Natural Science Division, Pepperdine University, Malibu, CA, United States

**Keywords:** folic acid, protein, exercise, physical activity, inflammatory biomarkers, neurocognitive behavior, fecal microbiota

## Abstract

Intestinal microbiota, diet, and physical activity are inextricably linked to inflammation occurring in the presence of tumor progression and declining neurocognition. This study aimed to explore how fecal microbiota, inflammatory biomarkers, and neurocognitive behavior are influenced by voluntary exercise and surplus dietary protein and folic acid which are common health choices. Dietary treatments provided over 8 weeks to C57BL/CJ male mice (*N* = 76) were: Folic Acid (FA) Protein (P) Control (FPC, 17.9% P; 2 mgFA/kg); Folic Acid Deficient (FAD); Folic Acid Supplemented (FAS; 8 mgFA/kg); Low Protein Diet (LPD, 6% P); and High Protein Diet (HPD, 48% P). FAS mice had decreased plasma HCys (*p* < 0.05), therefore confirming consumption of FA. Objectives included examining influence of exercise using Voluntary Wheel Running (VWR) upon fecal microbiota, inflammatory biomarkers C - reactive protein (CRP), Vascular Endothelial Growth Factor (VEGF), Interleukin-6 (IL-6), nuclear factor kappa ß subunit (NF-κßp65), Caspase-3 (CASP3), Tumor Necrosis Factor-alpha (TNF-α), and neurocognitive behavior. CRP remained stable, while a significant exercise and dietary effect was notable with decreased VEGF (*p* < 0.05) and increased CASP3 (*p* < 0.05) for exercised HPD mice. Consumption of FAS did significantly increase (*p* < 0.05) muscle TNF-α and the ability to build a nest (*p* < 0.05) was significantly decreased for both FAD and LPD exercised mice. Rearing behavior was significantly increased (*p* < 0.05) in mice fed HPD. An emerging pattern with increased dietary protein intake revealed more distance explored in Open Field Testing. At week 1, both weighted and unweighted UniFrac principal coordinates analysis yielded significant clustering (permanova, *p* ≤ 0.05) associated with the specific diets. Consumption of a HPD diet resulted in the most distinct fecal microbiota composition. At the phylum level–comparing week 1 to week 8–we report a general increase in the Firmicutes/Bacteroidetes ratio, characterized by an outgrowth of Firmicutes by week 8 in all groups except the HPD. MaAsLin2 analysis corroborates this finding and emphasizes an apparent inversion of the microbiome composition at week 8 after HPD. Explicit modification of oncogenic inflammatory biomarkers and fecal microbiome post high FA and protein intake along with voluntary exercise contributed to current underlying evidence that this diet and exercise relationship has broader effects on human health and disease–perhaps importantly as a practical modulation of cancer progression and declining neurocognition.

## Introduction

The advent of delineating dietary and exercise modulation for chronic diseases such as dementia and cancer is advancing through diligent investigations linking them with inflammatory processes and our intestinal microbiome. Non-communicable diseases have caused more than 80% of deaths in Western societies due to fluctuating dietary macronutrient choices impacting chronic disease progression (1). Global protein intake was 78.2 g/day in 2015, ranging from 61.0 g in the Middle East to 92.5 g/day in Asia. A popular macronutrient choice, chronic high protein intake (> 2 g per kg BW per day, ∼140 g/day for adults) may result in digestive and neurovascular abnormalities as mediated by the immune system (2, 3). Most humans may develop cancer or precancerous cells in their lifetimes and the level of dietary protein modulating this mechanism remains an elusive, conflicting, yet compelling factor. Indeed, lower rates of cancer in mice provided low-protein diets were observed, even after being implanted with 20,000 melanoma cells (4). Protein intake influences levels of the growth hormone IGF-1, which not only affects the growth of healthy cells, but also can encourage hyperplasia. One study found that for every 10 ng/ml increase in IGF-1, individuals consuming diets with 20% protein were 9% more likely to die from cancer than those on a lower (10%) animal-based protein diet (5).

An additional micronutrient essential for synthesis of protein *via* one carbon metabolism is the water soluble vitamin, folate. Low serum folate as clinically assessed by elevated levels of plasma homocysteine is considered a risk factor for neurocognitive diseases and oncogenesis (6, 7). Observational studies reported that high dietary intake of folate may possess some chemopreventive properties for cancer, whereas several studies described findings that chronic folic acid supplementation may actually increase this risk and maintain a low serum homocysteine (8–10). Despite contrary studies, there exists a narrow advantage of supplementation with folic acid for loss of learning memory in dementia (11). One study in Ts65Dn mice fed folic acid deficient diets documented shortened dendritic lengths in the dentate gyrus, cells that play a significant role in attention and contextual learning (12). In addition to protein and folate, a moderate, chronic intensity of exercise is necessary for an optimal inflammatory response as an efficient strategy for prevention of chronic disease (13, 14).

Due to enhanced physiological health, and improved neurocognition, exercise, alongside diet, present non-pharmacological approaches for the prevention of diseases whose pathophysiology is linked to sustained activation of the immune system (15). Emerging research suggests a positive influence of physical activity upon the inflammatory response such that it is becoming more evident that exercise may be a protective mechanism against colorectal cancer (CRC) and dementia (16, 17). Increased exercise stimulated a pro-inflammatory cytokine, namely interleukin-6 (IL-6) as well as vascular endothelial growth factor (VEGF) and induced expression of Caspase-3 (CASP3); although, it was noted that reduced cytokine concentrations occurred after prolonged physical activity (13). VEGF is a mediator of tumor angiogenesis and is expressed by most types of cancer (18, 19). CASP3 is often used as a measure of therapy efficacy as it modulates the migration, metastasis, and invasion of cancerous cells (20). In humans, it was observed that regular physical activity reduces plasma IL-6 and tumor necrosis factor-alpha (TNF-α) (21). Matsumoto et al. was the first to document that 5 weeks of exercise training resulted in an increase in the bacterial metabolite, butyrate (22). Whether this disease protection is mediated by exercise induced changes in the gut microbiome remains to be determined. A clear limitation of our study is that the data examines a narrow slice of the available cytokines and inflammatory biomarkers as localized in the liver and muscle. Furthermore, despite some significance among our inflammatory biomarkers, this data is not linked to our findings linking the diet and physical activity to the fecal microbiota.

An experimental model of physical activity, voluntary wheel running (VWR) has been shown to change the gut microbiota (23). Mika et al. revealed that microbial genera were more robustly altered by VWR in juvenile rats, whereas Huda et al. noted that VWR increased the microbial diversity in mice fed a high-fat diet (24). Evidently, an altered intestinal microbiome may be directly influenced by diet and host genetics as recognized by Huda et al., noting that C57BL/6J mice as used in our study, are more susceptible to altered gut microbiota compared to 3 other mouse strains (A/J, FVB/NJ, and NOD/ShiLtJ) (25). Modulation by exercise may release metabolites that may interact with the intestinal microbiome directly or indirectly through crosstalk with the immune system *via* the inflammatory response (26); although in the current study this crosstalk was not measured. In recent years, advancements have been made in understanding the involvement of the gut microbiome in neurocognitive diseases and in particular, CRC, as a regulator of substrate metabolism. Targeting host metabolism *via* the gut microbiome may therefore be a possible strategy to improve the effectiveness of diet and exercise interventions to promote neurocognitive health and modulate the prevention of cancer. In addition, an investigation of CRC biomarkers were compared between a meat- versus fish-based diet using the fecal microbiome as a determinant of the effect of diet on CRC risk (the MeaTlc study) (27). Studies linking dietary protein intake, fecal microbiome and its modulation of cancer development remain limited and inconclusive.

Inflammation is a biological response of the immune system that prevents, limits, and repairs damage by invading pathogenic or endogenous molecules, and the intestinal microbiome of an organism complements and interacts in this response. Once activated, through dietary and exercise choices, the immune system responds by releasing cytokines classified as measurable pro-inflammatory (TNFα, CRP, NF-kβp65, VEGF, CASP3) or anti-inflammatory (IL-6). Chronic exercise mediates this inflammatory response which may be important for our health, mainly warding off chronic disease. The aim of this study was to investigate: (1) the role of folic acid, protein, and exercise upon neurocognitive behaviors associated with loss of attention and contextual learning (Nest building, Open Field Tests (OFT), Novel Object Recognition (NOR); (2) the role of folic acid, protein, and VWR (exercise) upon the cytokine response and oncogenic biomarkers as an anti-inflammatory response perhaps experienced in chronic diseases; and, (3) the role of folic acid, protein, and VWR impact upon the fecal microbiota.

## Materials and methods

### Animals and experimental design

**FIGURE 1:**
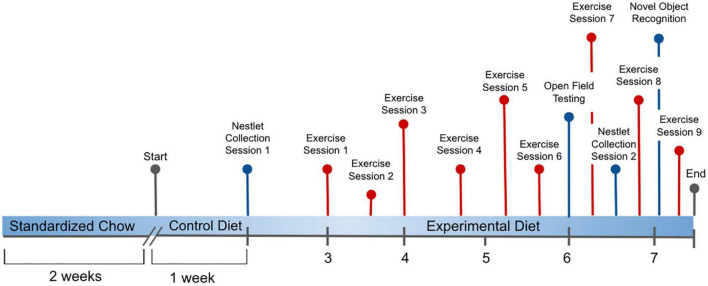
Experimental process initiated with all C57BL/6J male mice provided *ad libitum* to a standardized Chow diet (Teklad, Envigo #8604, 24.3% protein) for 2 weeks, followed by provision of the Folic Acid (FA) Protein (P) Control diet [Harlan Custom Research Diet (TD 15201), FPC, 2 mg/kg folic acid, 17.9% protein] for 1 week. From weeks 2 to 8, mice were distributed across these 5 experimental diets: (1) 0 mg/kg folic acid FA/17.9% protein, TD.150200, FA Deficient (D) Diet (FAD; *n* = 16); (2) 2 mg/kg FA/17.9% protein; TD.150201, Control (C) FA Protein (P) Diet (FPC; *n* = 12); (3) 7 mg/kg, FA/17.9% protein; TD.15202, FA Supplemented (S) Diet (FAS, *n* = 16); (4) 2 mg/kg FA, 6% protein [Low Protein Diet (LPD, *n* = 16)]; and (5) 2 mg/kg FA/48% protein [High Protein Diet (HPD, *n* = 16)]. Nestlet collection occurred at weeks 1 and 6.5. Open Field Testing was assessed at week 6, and Novel Object Recognition was evaluated during week 8. Voluntary Wheel Running of the mice measured exercise during 9 sessions over 5 weeks.

Twelve to fourteen weeks old C57BL/6J male mice (The Jackson laboratory, #000664) were group-housed (*n* = 4) in standard plastic cages and maintained in a temperature controlled environment 22°C on a 12-h light-dark cycle. Each cage was enriched with tunnels and a Mouse House*™* (Techniplast, red polycarbonate, L50 x W 110 × H 77 mm), designed to enhance positive behavior and increase visibility for maintaining care of the mice. Mice were divided into nineteen groups for housing, then randomly assigned to five dietary treatments. The experimental design is illustrated in Figure 1.

Mice were provided standard rodent chow, (Teklad, Envigo #8604, 24.3% protein) for 2 weeks with provision of the custom folic acid protein control [FPC, Harlan Custom Research Diet (TD)] diet for 1 week, and experimental diets offered at ages 14–15 weeks, *ad libitum*. Duration of experiment was 8 weeks with mice provided the following custom dietary treatments (Table 1): (1) 0 mg/kg folic acid FA/17.9% protein, TD.150200, FA Deficient (D) Diet (FAD; *n* = 16); (2) 2 mg/kg FA/17.9% protein; TD.150201, Control (C) FA Protein (P) Diet (FPC; *n* = 12); (3) 7 mg/kg, FA/17.9% protein; TD.15202, FA Supplemented (S) Diet (FAS, *n* = 16); (4) 2 mg/kg FA, 6% protein [Low Protein Diet (LPD, *n* = 16)]; and (5) 2 mg/kg FA/48% protein [High Protein Diet (HPD, *n* = 16)].

**TABLE 1:** Concentration of folic acid (mg/kg) and percent protein in five different dietary treatments provided *ad libitum* to C57BL/6J mice (The Jackson Laboratory, #000664) for 8 weeks and the concentration of plasma homocysteine (ug/mL).

Dietary treatment	*n*	Folic acid, mg/kg	Protein, (%)	Fat, (%)	CHO, (%)	Plasma homocysteine			
						*n*	ug/mL	SEM	
Folic Acid/Protein Control (FPC)	12	2	19.2	14.5	66.2	7	0.902	0.067	
Folic Acid Deficient (FAD)	16	0	19.2	14.5	66.2	12	0.890	0.063	
Folic Acid Supplemented (FAS)	16	8	19.2	14.5	66.2	15	0.429	0.038	*p* < 0.05*
Low Protein Diet (LPD)	16	2	6.8	14.6	72.9	12	0.862	0.098	
High Protein Diet (HPD)	16	2	52.0	14.5	33.5	12	0.809	0.081	

*Significance of Folic Acid Supplemented (FAS) plasma homocysteine from *t*-test, two-tailed analysis.

Routinely, mice were weighed individually on a Sartorius balance (BCE124l1S), bedding (Envigo, Teklad, 1/8” Corncob) was replaced, and food and water provided *ad libitum*. At the end of the study, animals were deeply anesthetized using Nembutal^®^ sodium solution CII (Oaks Pharmaceuticals, Inc., Lake Forest, IL, USA, NDC: 7648-501-20). Blood was collected by cardiac puncture in potassium-EDTA coated 1.7 mL polypropylene centrifuge tubes and plasma was separated, then tissues were collected in liquid nitrogen and stored in −80°C until biochemical analyses were carried out. All animal handling procedures were approved by the Pepperdine University IUCAC (#05211).

### Measurement of plasma homocysteine

To confirm adequate consumption of folic acid, plasma homocysteine was measured (Table 1). Blood was collected by cardiac puncture in potassium-EDTA coated 1.7 mL polypropylene centrifuge tubes and plasma was separated by centrifugation (16,000 × *g*, 20 min) and stored at −80°C. Soluble aminothiols were derivatized with SBD-F (ammonium 7-fluorobenzo-2-oxa-1,3-diazole-4-sulfonate, Fisher Scientific, Waltham, MA, USA) to produce a fluorescent adduct prior to HPLC-FLD analysis using a method adapted from the literature (28). A 50 μL aliquot of plasma was mixed with 10 μL of tri-*n*-butylphosphine (TBP, 100 μL mL^–1^ in DMF), and incubated at 4°C for 30 min. Proteins were then precipitated with the addition of 50 μL of trichloroacetic acid solution (10% w/v TCA, 1 mM EDTA) and incubating at room temperature for 10 min. The samples were centrifuged for 10 min (4°C, 16,000 × *g*). A 50 μL aliquot of the supernatant was transferred to a new 1.7 mL centrifuge tube containing 10 μL of 1.55 M NaOH, 200 μL of 125 mM borate buffer with 4 mM EDTA (pH 9.5), and 50 μL of SBD-F solution (1.0 mg mL^–1^ in 125 mM borate buffer, pH 9.5). The samples were incubated at 60°C for 1 h and cooled on ice.

### Preparation of calibration standards

Calibration standards were prepared by suitable dilution of a more concentrated stock solution. Standards were treated identically as samples. The concentration of the standards at the point of injection ranged from 0.45 to 51.3 μM. Standard solutions were stored at −10°C when not in use and prepared fresh weekly.

### HPLC-FLD analysis

Analysis of standards and samples was performed by reversed-phase high performance liquid chromatography coupled with fluorescence detection using a Luna C18 column (4.6 × 150 mm, 5 μm *d*_*p*_, Phenomenex, Inc.) protected with a guard cartridge (SecureGuard^®^, Phenomenex). The HPLC instrument consisted of a Shimadzu LC-20A system equipped with a ternary pump (LC-20AD), refrigerated autosampler maintained at 4°C (SIL-20AC), and fluorescence detector (RF-20A). The separated aminothiol derivatives were detected with an excitation wavelength of 385 nm and an emission wavelength of 515 nm. Isocratic separation was performed using 99% 100 mM acetate buffer (pH 5.0)/1% acetonitrile at a flow rate of 1 mL min^–1^. Injection volume was 10 μL. HPLC solvents were vacuum degassed continuously throughout analysis.

### Physical activity

Voluntary wheel running (VWR) was used to model exercise training as an adaptive inflammatory response to dietary changes. During the 12-h light cycle, mice from the physical activity group were individually housed, in an unfamiliar setting, to ensure accurate reading of physical activity for each animal. Each mouse was assigned a designated running wheel prior to experiment commencement. The cage was lined with fresh bedding, with 1–2 pellets of the assigned diet, and fresh water from a water bottle. Before mouse exposure, the Actimetrics Wireless Low-Profile Angled Running Wheel (Model 86180) by Lafayette Instrument (Lafayette, IN) was placed in each cage and locked using a lock pin. After the 12-h acclimation period, the wireless connection was confirmed and respective lock pins were removed from each wheel. Collectively the wheels were turned “on” to allow for continuous data collection using the Actimetrics ClockLab Data Collection System (Model 86187), and the mice were allowed *ad lithium* access to the wheel for the entirety of the dark cycle (12 h). Each wheel used was calibrated to relay data from the wheel rotation sensor to the Actimetrics Wireless USB Gateway (Model 86185), collecting the number of wheel rotations every 30 s.

This data was then transferred to the Actimetrics ClockLab Analysis Application (Model 86188) for analysis and generation of actograms and periodograms. At the completion of the 12-h dark cycle, the mice were removed from the setting and returned to experimental cages. This procedure was repeated biweekly during weeks 5 to 8 for a total of 9 sessions of physical activity for each mouse.

### Behavioral measurements

Overnight (12 h) mice were individually placed into a new environment (different size cage), provided water, designated diet, a nestlet (Ancare, NES3600), and sufficient bedding. The following day, nestlet remains were photographed. Nestlets were scored on a scale of 1–5 (29, 30). A Nestlet building score of 1 indicated the nestlet was largely untouched, a score of 2 indicated the nestlet was partially torn, and a score of 3 indicated the nestlet was mainly torn however no clear nest site. The more efficient nest building had a score of 4 indicating there is an evident yet flat nest and a score of 5 indicating the mouse tore up the entire nestlet and created a mound-shaped nest and was submerged within the nest (31). For Open Field Testing (OFT), mice were singly transported to the lab in a covered cage. Mice were then housed in the lab room for 30 min in their home cage lined with bedding, with food and water *ad libitum*. After a 30 min acclimation phase, the mice were placed into the center of the open field arena (60 cm × 60 cm × 60 cm) with white bottom and black walls and were allowed to freely explore the arena for 30 min. A 5 cm wide border margin was defined as the corridor, and the inner square of 40 cm × 40 cm included in the center of the arena. The number of rears and total distance traveled were recorded for 30 min using ANY-maze video tracking software (Stoeltling Co., Wood Dale, IL, USA), and the photobeam which was preset to 75% of the sample mean length to register rearing activity. In between evaluations, the open field arena was thoroughly cleaned with 70% ethanol solution after each trial to reduce traces of previous mouse exploration. Novel Object Recognition (NOR) tests were performed according to previously established protocol with minor modifications (32). The NOR test consisted of 3 sessions: habituation, training, and retention. Each mouse was individually habituated to a plastic open arena (60 cm × 60 cm × 60 cm) during the NOR test for 3 days. One day before testing, the mice were isolated individually into separate habituation quadrants (40 cm × 40 cm) for a 10 min environmental habituation period. After 10 min, the mice were placed back into the cage. After an interval of 24 h, the mice were placed into the familiarization quadrant containing 2 identical objects for 10 min. Each of the objects was placed in two neighboring corners of the quadrant. During the training session, 2 identical objects were placed into the opposing corners of the center of the arena 30 cm apart from one another and mice were allowed to explore for 5 min. Exactly 1 h later, a common training to testing interval for robust recognition during the retention session, the mice were reintroduced into the test quadrant which now contained 1 novel object and 1 familiar object for 5 min, the positions of the objects were changed in between each test to avoid bias. The number of approaches, rearing, and the time each mouse spent exploring each object were recorded. Interaction with the object was defined as the nose facing the object within 2 cm or less. Climbing or sitting on the object was not recognized as interaction with the object. Time spent exploring the objects during the trials was determined. All movement of the mouse was filmed using ANY-maze video tracking software, and a photobeam was placed alongside the testing arena to register rearing activity for the duration of the NOR test. Quadrants in the arena and all objects were thoroughly cleaned with 70% ethanol solution after each trial.

### Inflammatory biomarkers

The inflammatory biomarkers were assessed from individual Abcam (Canada) ELISA assay kits. Liver concentration of C-Reactive Protein (CRP, ab222511), Interleukin 6 (IL-6, ab100713), Vascular Endothelial Growth Factor, (VEGF, ab209882), Caspase-3 (CASP3, ab39401), Nuclear Factor kappa beta p65, (NF-kβp65, ab176663), and muscle concentration of Tumor Necrosis Factor, (TNF-α, ab285327), were analyzed with slight modifications of the manufacturer instructions. A total of 100 μl standard sample and 100 μl diluted sample were added to the reaction plate and incubated at 37°C for 30 min. After washes, 100 μl tested sample was added to each well and then incubated at 37°C for 2 h. After washes, 100 μl horseradish peroxidase-labeled secondary antibody was added to each well. Samples were incubated at 37°C for 30 min. After washes, developer A and developer B, each 50 μl, was added. The tested sample was developed for 15 min in the dark. A stop solution was added at 50 μl per well to terminate the reaction. Optical density at 450 nm was read using a microplate reader (Biotek-Multiscan Elx808iu; Gen5 software). A standard curve was drawn. According to the curve equation, the concentration of the corresponding sample was calculated and averaged across samples.

### Fecal microbiota analysis, (1, 4, 8 week)

DNA Isolation and 16S library preparation: Fecal samples (DNeasy Powersoil Pro kit, QIAGEN) were submitted to the UC Davis Host Microbe Systems Core for DNA isolation, library preparation, and sequencing. The V3-V4 region of the 16S rRNA gene was amplified and indexed *via* PCR using primers (primers 319F and 806R). No template controls were included in all PCR steps and these controls yielded no amplification. Amplicons were quantified and pooled to equalize concentrations for Illumina MiSeq bidirectional paired-end sequencing (2 × 300 bp; Illumina, San Diego, CA, USA).

### Sequence preprocessing

Overlapping paired-end reads were processed into amplicon sequence variants (ASVs) using the DADA2 software (33). Forward reads were trimmed to 265 bp and reverse reads were trimmed to 185 bp. This was the only modification made to the default DADA2 pipeline. All 120 samples were sequenced in the same run. However, the input amount for the first 80 samples was higher than the last 40. For this reason, the first 80 samples yielded an average of 56,000 reads that could be assigned a taxonomy, while 40 samples yielded only 22,000 reads, on average, that could be assigned a taxonomy. All fastq files for the microbiome data can be found at: Sequence Read Archive: Accession ID: PRJNA875949.

### Analysis of microbiota composition

Raw ASVs and taxa used in this analysis are available as Supplementary file. All R source code can be found in Supplementary file. All non-bacterial reads were removed and the data was rarefied to the smallest sample depth of 15,578 reads. We used the package APE in R to construct a phylogenetic tree (34). Next, we used the *phyloseq* and *vegan* packages in R to analyze alpha (based on observed ASVs, Chao1, and Shannon Diversity Index) and beta diversity [weighted and unweighted unique fraction metric (UniFrac)] principal coordinates analysis, and relative phylum and family abundance (35, 36). Analysis of alpha and beta diversity was conducted on the rarefied dataset using all ASVs. The betadisper test in *vegan* was applied to determine relative heterogeneity of the different diet microbiota. This function analyzes the variation of each sample from the centroid. A *p* ≤ 0.05 from betadisper suggests significant dispersal of the microbiota between diets. Betadisper returned only non-significant results for all comparisons (*p* ≥ 0.05), meriting the use of PERMANOVA to compare the sample clustering scheme. PERMANOVA was then used to compare clustering of samples. A *p* ≤ 0.05 suggests the clustering scheme on the PCoA is significant. We used the Shapiro-Wilk test for normality on the alpha diversity metrics, with a *p* value ≥ 0.05 indicating that the samples were normally distributed. Based on the test for normality, we applied repeated-measures analysis of variance (ANOVA) and *post hoc* Tukey’s tests to determine statistical significance between diets. Prior to analysis of relative abundance we filtered out ASVs with fewer than 20 reads from the un-rarefied dataset. ASVs were transformed to percent abundance prior to analysis of relative phylum and family abundance and MaAsLin2 We analyzed percent abundance using all ASVs at the phylum and family level. All plots were created using *ggplot2* (37). MaAsLin2 is a multivariate statistical model that identifies associations between microbial features and metadata (38). MaAsLin2 was used under default settings with a *q* value threshold of 0.25 (Benjamini-Hochberg adjustment) to identify differential abundant ASVs associated with diet (38).

### Statistical analysis

All values are reported as mean ± SEM. The differences between two groups were analyzed using Student’s *t*-test. Body weights, physical activity, and behavioral measurements were analyzed with repeated measures two-way ANOVA, and Tukey’s HSD *post hoc* test was applied when appropriate. Inflammatory biomarker data analyses were conducted using GraphPad Prism version 8.0 available for Windows (GraphPad Software, San Diego, CA, USA) and SPSS Statistics 26 (IBM Corporation, Armonk, NY, USA). The *p* < 0.05 denotes statistical significance.

## Results

**FIGURE 2:**
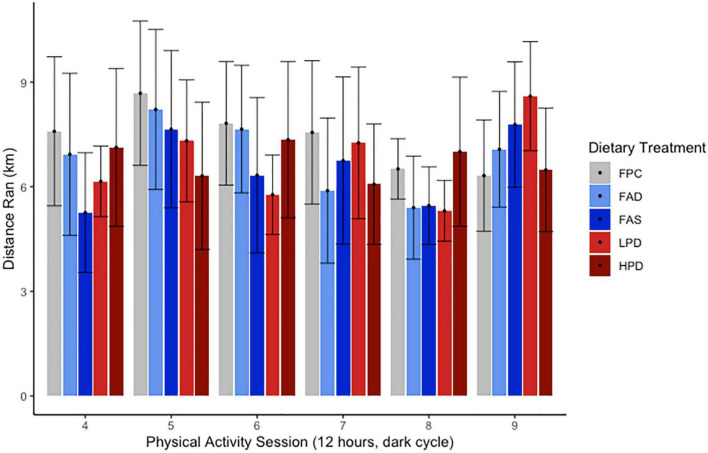
Effect of dietary treatments upon distance run (km) over 12 h during 6 sessions over experimental weeks 5–8 of Voluntary Wheel Running per mouse. C57BL/6J mice were provided these 5 experimental diets: (1) 0 mg/kg folic acid FA/17.9% protein, TD.150200, FA Deficient (D) Diet (FAD; *n* = 16); (2) 2 mg/kg FA/17.9% protein; TD.150201, Control (C) FA Protein (P) Diet (FPC; *n* = 12); (3) 7 mg/kg, FA/17.9% protein; TD.15202, FA Supplemented (S) Diet (FAS, *n* = 16); (4) 2 mg/kg FA, 6% protein [Low Protein Diet (LPD, *n* = 16)]; and (5) 2 mg/kg FA/48% protein [High Protein Diet (HPD, *n* = 16)]. The results are not significant at *p* < 0.5.

A dietary intervention of 8 weeks with deficient or supplemented folic acid or protein did not result in a significant change in body weights of all mice. Routine measurement of body weights over 8 weeks ranged from 17.6 ± 3.24 to 24.6 ± 2.57 g at week one and, at the end of the experiment, 30.6 ± 3.23 to 32.1 ± 2.76 g. Gradual growth occurred for all mice as they were ages 12–14 weeks at start of experiment and then 23–25 weeks by end of the acclimation to chow/control diet (3 weeks) and experimental period (8 weeks). No significant difference was measured among the five dietary treatments in rate of growth. Though the dietary composition differed in protein and carbohydrate (Table 1), the percentage of fat and calories were isocaloric. According to Figure 2, the VWR over 8 weeks revealed no discernable pattern or significance among the different dietary interventions or deficient/supplemented folic acid and low/high protein. In general, and without statistical significance, a pattern emerged of more distance traveled for the FAD, FAS, and LPD mice, with a minor increase of distance for the HPD. The different folic acid and protein dietary levels provided to the VWR C57Bl mice apparently had no effect on the distance traveled between the end of the period and the beginning of the exercise period.

### Effects of folic acid and protein on neurocognitive behavior

**FIGURE 3:**
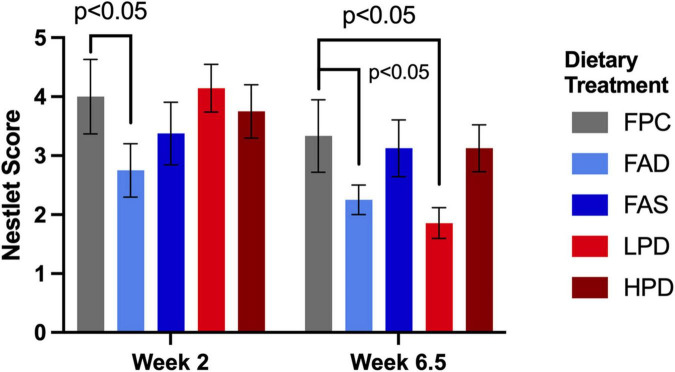
Effect of dietary treatment upon nestlet building as measured by a Nestlet Score of 1 to 5 at weeks 2 and 6.5. C57BL/6J mice were provided these 5 experimental diets: (1) 0 mg/kg folic acid FA/17.9% protein, TD.150200, FA Deficient (D) Diet (FAD; *n* = 16); (2) 2 mg/kg FA/17.9% protein; TD.150201, Control (C) FA Protein (P) Diet (FPC; *n* = 12); (3) 7 mg/kg, FA/17.9% protein; TD.15202, FA Supplemented (S) Diet (FAS, *n* = 16); (4) 2 mg/kg FA, 6% protein [Low Protein Diet (LPD, *n* = 16)]; and (5) 2 mg/kg FA/48% protein [High Protein Diet (HPD, *n* = 16)]. The results are means ⨦ SEM (*n* = 8). *p* < 0.05, week 6.5, FPC vs. LPD. Significance at *p* < 0.05, week 2 and 6.5, FPC vs. FAD.

Mice require less than 10 min to build a nest (39), and standard experimental protocol allows nest building for the duration of the 12 h dark period. Similar to a previous study’s findings, the folic acid deficient mice in both weeks 2 and 6.5 were significantly less capable of building a nest than the mice fed adequate folic acid and protein, *p* < 0.05 (12). Mice performed nest building activity during week 2 as they were acclimating to the experimental diets and at week 6.5, toward the end of the study. Indeed, at week 6.5, the mice consuming the low protein diet became significantly less able to construct a nest (*p* < 0.05) as compared to the mice with adequate and supplemented folic acid than mice fed the higher protein diet (Figure 3).

In OFT, an analysis of variance showed that the total distance (m) traveled was not statistically significant among mice in the five dietary groups. Additional analyses of variance showed that there were no statistically significant differences in periphery time (s) rearing line crossings, or rearing time (s) among mice in the five dietary groups. The HPD mice had more distance traveled with less variability than any other dietary treatment, although not statistically significant. In NOR testing, there was statistically significant difference in rearing between FPC and HPD mice (*p* < 0.05), and significance with novel object exploration (*p* < 0.05) among mice in the five dietary groups, with LPD mice exploring less (Table 2).

**TABLE 2:** Rearing and novel object exploratory behavior assessed in C57BL/6J mice over 7 weeks provided five different experimental diets *ad libitum*.

Dietary treatment	Rearing behavior			Novel object exploration	
	*n*	Mean	SEM	Mean	SEM
Folic Acid Control (FPC)	6	15.17	1.558	65.00	2.191
Folic Acid Deficient (FAD)	6	14.17	2.688	61.67	2.917
Folic Acid Supplemented (FAS)	6	16.33	2.753	64.83	3.790
Low Protein Diet (LPD)	6	13.83	1.222	58.17*	2.725
High Protein Diet (HPD)	6	19.33*	2.487	63.67	2.929

Rearing behavior consisted of the number of times mouse standing on both hind paws in a vertical upright position while tracked using an infrared beam grid raised 6.5 cm above the locomotion grid. Novel object exploration was the number of times the mouse approached the novel object randomly placed in an open arena.

*Significance (Tukey HSD), *p* < 0.05.

### Effects of folic acid, protein, and exercise on inflammatory biomarkers

**FIGURE 4:**
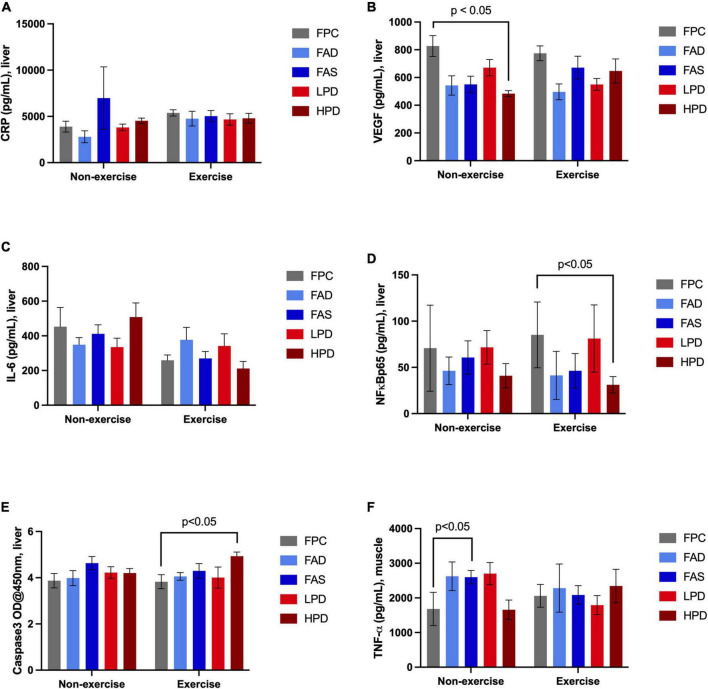
Effect of dietary treatment and influence of exercise upon inflammatory biomarkers in liver and muscle in C57BL/6J mice. Liver concentration of **(A)** C-Reactive Protein (CRP, pg/mL); **(B)** Vascular Endothelial Growth Factor (VEGF, pg/mL); **(C)** Interleukin 6 (IL-6, pg/mL): **(D)** NF kappa ß p65, (pS536 + Total), pg/mL; **(E)** Caspase-3 (OD@450 nm); and, muscle concentration of **(F)** Tumor Necrosis Factor, (TNF-α, pg/mL). Mice were provided these 5 experimental diets: (1) 0 mg/kg folic acid FA/17.9% protein, TD.150200, FA Deficient (D) Diet (FAD; *n* = 16); (2) 2 mg/kg FA/17.9% protein; TD.150201, Control (C) FA Protein (P) Diet (FPC; *n* = 12); (3) 7 mg/kg, FA/17.9% protein; TD.15202, FA Supplemented (S) Diet (FAS, *n* = 16); (4) 2 mg/kg FA, 6% protein [Low Protein Diet (LPD, *n* = 16)]; and (5) 2 mg/kg FA/48% protein [High Protein Diet (HPD, *n* = 16)]. The results are means ⨦ SEM (*n* = 8). Panel **(B)** VEGF *p* < 0.05, exercised FPC vs. HPD; panel **(D)** NF kappa ß p65 *p* < 0.05, exercised FPC vs. HPD; panel **(E)** Caspase-3 *p* < 0.05, exercised FPC vs. HPD; and, panel **(F)** muscle TNF-α, unexercised FPC and FAS, *p* < 0.05.

Examination of chronic inflammation associated with proliferation of cancer was assessed by a limited number of liver (*n* = 5) and muscle (*n* = 1) inflammatory biomarkers in response to exercise and dietary interventions (Figure 4). Typically, the longer inflammation persists the greater the risk of cancer. No noticeable change in liver CRP response measured over 8 weeks of dietary interventions nor with additional exercise. Expressed by most types of cancer as a consequence of chronic inflammation is Vascular Endothelial Growth Factor (VEGF), which significantly decreased (*p* < 0.05) after mice consumed a high protein diet; and though not statistically significant, a similar pattern was observed post VWR after 8 weeks. A cytokine stimulated by contracting skeletal muscles during exercise, hyperhomocysteinemia, and involved in the control of cell proliferation and apoptosis is Interleukin 6 (IL-6). Although not statistically significant, there was a noticeable decrease in IL-6 in the exercised mice, in particular in the FAS and HPD mice. VFW did stimulate an increase in NF-kβp65 though significantly between FPC and HPD exercised mice. In fact, the mice fed HPD had reduced NF-kβp65 as compared to mice allowed adequate folic acid and protein. CASP3 modulates the migration, metastasis, and invasion of cancerous cells. Mice consuming HPD while exercising for 8 weeks had significantly (*p* < 0.05) increased CASP3. Muscle TNF-α is a contributor to NF kβp65 activation and this pattern persisted in analysis of TNF-α in the HPD mice, though not statistically significant.

### Fecal microbiota analysis

**FIGURE 5:**
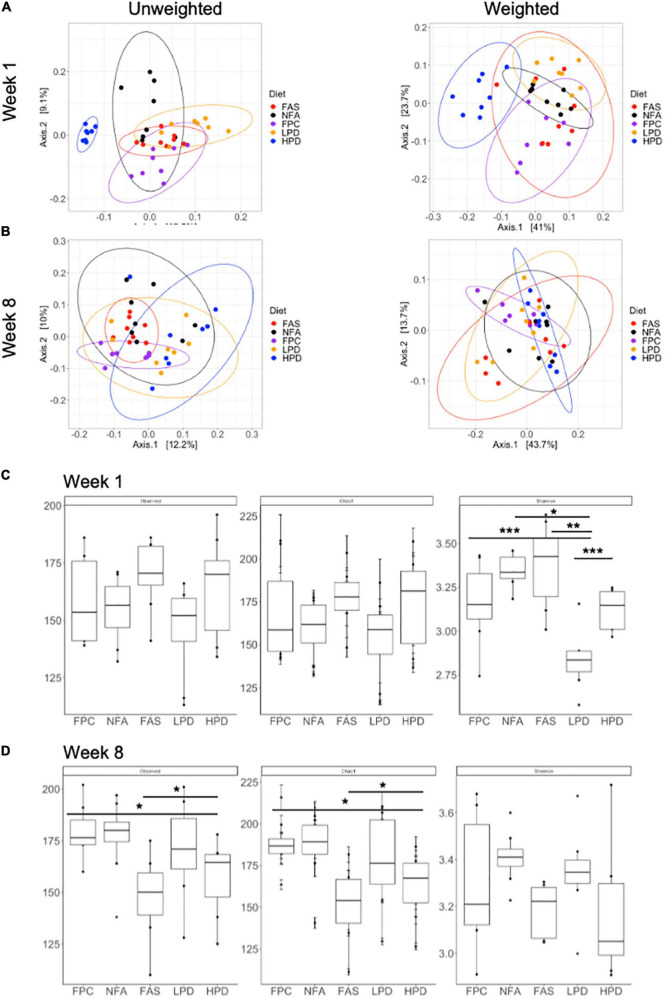
Alpha and beta diversity of the fecal microbiota across diets at weeks 1 and 8. **(A)** Unweighted unique fraction metric (UniFrac) and weighted UniFrac principal coordinates analysis (PCoA) at week 1 (*p* ≤ 0.05) and **(B)** week 8 (*p* ≤ 0.05) across all groups (FPC, FAS, NFA, LPD, and HPD). FPC is the control group. *p* ≤ 0.05 suggests the clustering scheme across all groups is significant. There is no *post hoc* test for PERMANOVA. Alpha diversity (Observed, Chao1, and Shannon Diversity Index) of the fecal microbiota at **(C)** week 1 (Shannon diversity: *p* < 0.05 NFA relative to HPD and LPD; *p* < 0.01 FAS relative to LPD; *p* < 0.001 FPC relative to LPD and LPD relative to HPD) and **(D)** week 8 (Observed ASVs *p* < 0.05 FPC relative to HPD and FAS relative to HPD; Chao1: *p* < 0.05 FPC relative to HPD and FAS relative to HPD). FPC is the control group *p ≤ 0.05; **p ≤ 0.01; ***p ≤ 0.001.

Global diversity of the fecal mouse microbiota was analyzed at weeks 1 and 8 (at the start and end of the experiment) using alpha and beta diversity metrics. At week 1, both weighted and unweighted UniFrac principal coordinates analysis yielded significant clustering (permanova, *p* ≤ 0.05) associated with the specific diets. When the relative abundance of specific bacterial taxa is not considered (unweighted), these clusters become even more distinct. In particular, the HPD diet results in the most distinct fecal microbiota composition, relative to the other diets (Figure 5A). At week 8, permanova still suggests significant clustering of the fecal microbiota samples (*p* ≤ 0.05). However, the LPD and HPD mice microbiotas appear more similar than at the start of the experiment at week 1 (Figure 5B). Additionally, at week 8, the FPC (control) microbiota appears more similar in composition to the FAS and NFA microbiotas (Figure 5C). Alpha diversity was analyzed according to three metrics, observed ASVs, Chao1, and Shannon Diversity (Figure 5B). In particular, significant shifts were noted in alpha diversity according to the Shannon Diversity Index between diets, which takes into account both richness and distribution of bacterial taxa in the microbial community. Of note, these shifts are no longer apparent at 8 weeks, suggesting similar diversity of the microbiota between mouse diets (Figure 5D).

**FIGURE 6:**
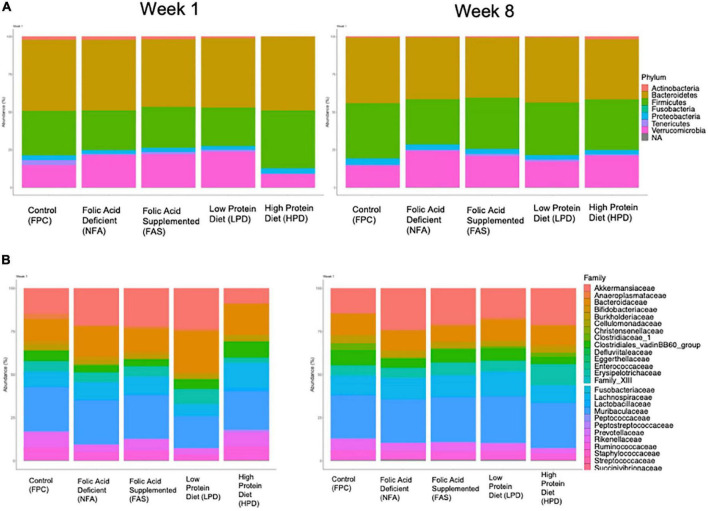
Relative **(A)** phylum and **(B)** family abundance of the fecal microbiota across diets. All ASVs were included in an analysis of percent phylum and family level abundance across diets at weeks 1 and 8. Colors correspond to taxa represented in each legend and the size of the boxes on the bar graphs indicates relative percent abundance of that taxon.

We aggregated ASVs at the phylum and family level for an overview comparison of the fecal mouse microbiota across diets. Similar to the analyses of alpha and beta diversity, after 8 weeks of supplementation, the phylum and family level microbiota appear more similar in composition across diets (Figure 6). At the phylum level, both the FAS and HPD favor an outgrowth of Verrucomicrobia by week 8 (Figure 6A). This is likely driven by an increased abundance of Akkermansiaceae. In addition, relative to controls, in week 8 the HPD and FAS resulted in reduction of Clostridiales_vandinBB60_group relative to the control group (Figure 6B).

**FIGURE 7:**
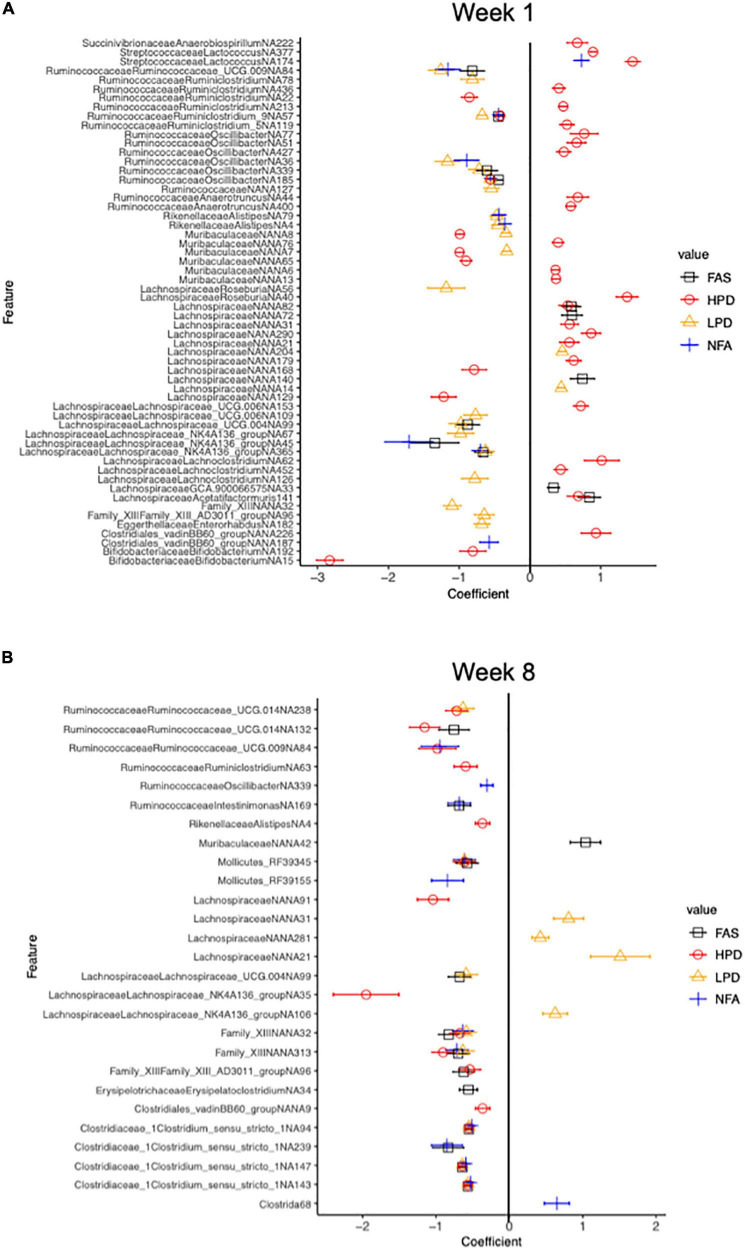
Differential relative abundance analysis relative to the control diet group (FPC) at 1 week and 8 weeks. Counts were transformed to relative abundance prior to analysis. The top 50 and top 52 ASVs are reported in panels **(A,B)**, respectively. ASVs in panel **(A)** all have a *q* value less than 0.006 and in panel **(B)**, a *q* value less than 2.53e-0.02 (Benjamini-Hochberg adjustment). The control (FPC) group is used as the reference group with FAS, HPD, LPD, and NFA being compared to this group. ll coefficients for these groups are relative to 0. A negative coefficient implies a decreased abundance of that taxon and a positive coefficient implies an increased abundance of that taxon.

We used MaAsLin2 to identify differentially abundant ASVs associated with the mouse diet. MaAsLin2 identified 397 differentially abundant ASVs at week 1 and 232 differentially abundant ASVs at week 8. At week 1, FAS and HPD displayed increased abundances of numerous taxa relative to the control group (FPC), particularly in the Lachnospiraceae family (Firmicutes phylum) (Figure 7A). Interestingly, by week 8, both of these supplements showed a marked decrease in these taxa relative to controls (FPC) (Figure 7B). Collectively, these data highlight significant shifts in microbiota composition after supplementation with folic acid and increased protein intake in mice.

## Discussion

Intestinal microbiota, diet, and physical activity are inextricably linked to the inflammatory response associated with tumor progression and declining neurocognition. In this study, VWR was used, rather than forced treadmill running (FTR) as it is known that FTR alters the immune process and mortality rate in C57BL/6J mice (40). Our study examined young adult mice introduced to isocaloric deficient or supplemented folic acid and provided low and high protein diets (Table 1); and, if our focus had been content of carbohydrate, low and high carbohydrate diets, as this nutrient was counterbalanced to create our low and high protein diets. Instead, this study questioned the role of two nutrients of importance to cellular growth and differentiation, folic acid and protein as they are essential to slow tumor progression and prevent neurocognitive decline. Supplementation of folic acid is notably the micronutrient source demonstrated to improve memory in sufferers of early neurocognitive decline, such as dementia (41, 42). Mice provided the folic acid deficient diets had diminished nest building ability after both 2 and 6.5 weeks; an expected outcome given an earlier experiment with Ts65Dn mice with same diet composition from Envigo (12). Additionally, the physiological and metabolic peril of under consumption of protein presented in our mice as significantly reduced nest building and less time spent exploring the novel object, both measures indicating apparent decline of neurocognitive function (43, 44). Evidently, despite varying folic acid and protein intake, the rearing behavior exhibited by all mice did not significantly differ, with the exception of the HPD mice exhibiting significantly increased rearing (*p* < 0.05) (Table 2). Rearing as measured by OFT has been associated with changes in the locus coeruleus cells of the hypothalamus in Alzheimer models of mice (45, 46). Consuming diets deficient in folic acid and protein did alter the ability of our mice to routinely build a nest and they displayed less curiosity during exploration of a new environment, both measures indicative of neurocognitive decline.

Our mice were exercised by VWR for 8 weeks, without significant differences in distance run in the 12 h session despite provision of varying levels of dietary folic acid and protein. Since isocaloric diets were provided and there was a shorter duration of VWR (8 weeks), then it is not surprising that a differentiated pattern of physical activity level was not specifically observed. Instead, using this baseline of physical activity among the varying dietary treatments allowed a model to measure the impact of the diet upon both the inflammation response pre and post chronic exercise. In this study, both liver (CRP, VEGF, IL-6, NF kβp65, CASP3) and muscle (TNF-α) inflammatory biomarkers were assessed. CRP is an acute-phase systemic protein produced primarily in the liver in response to stimulation by IL-6. Besides studies demonstrating consistently an increased risk of mortality due to inflammation and subsequent cancer development, both CRP and IL-6 have also been shown to be associated with non-cardiovascular mortality (47). Intestinal inflammation is also known to have an intrinsic role in the pathogenesis of colorectal cancer (48). Likewise, sustained neuroinflammation is a risk factor for dementia, and is increasingly considered a culprit of both frailty and disease in older aged individuals. Chronic moderately elevated expression of CRP is associated with an increased risk of a wide range of diseases (49). In this study, for both the unexercised and exercised mice, the CRP remained stable with low baseline concentrations, perhaps a protective effect of both diet and sustained physical activity. Yet, another pro-inflammatory cytokine, VEGF, is associated with exercise-induced adult hippocampal neurogenesis and tumorigenesis in colitis-associated cancer (50, 51). In our study, the unexercised mice that consumed high dietary protein displayed increased VEGF, an adverse impact. In contrast, folic acid supplementation and high protein intake by our mice, alongside 8 weeks of VWR resulted in a decreased IL-6, a protective effect. This finding aligns with a study demonstrating that moderate intense exercise can modulate IL-6 by lowering its basal levels and by causing lower acute increases in the plasma levels in response to exercise (52). Similarly, in another study about attenuating muscle loss in diabetic db/db mice, there was marked suppression of IL-6, TNF-α and NF-kβp65 post moderate exercise training (53). In contrast to these studies, a 2004 study questioned if IL-6 might be an exercise induced factor since the IL-6 gene is rapidly activated during exercise (54). Sometime later, in 2018, a systematic review of the epidemiological evidence revealed an association of higher concentrations of circulating IL-6 in cancer patients than healthy controls (55). Taken together, it may be that certain dietary choices, such as increased folic acid and protein, may modulate IL-6 in the presence of physical activity suppressing the negative consequences of chronic inflammation leading to chronic disease. Indeed, it should be noted that a constraint of this current data is that it is only a measurement of the impact of dietary treatment and VWR on a limited number of inflammatory biomarkers. Another potential modulator of tumor development is NF-kβp65, a sequence-specific transcription factor that is known to be involved in the inflammatory and innate immune responses. In our experiment, both exercise and high protein intake reduced NF-kβp65 (56, 57). CASP3 is a regulator of the migration, invasion, and metastasis of colon cancer cells. As such, many anticancer therapies are able to cause tumor cell death by activating CASP3, employing this proteolytic activation as it may increase efficacy of cancer treatment (20, 58). Both exercise and a high dietary protein intake increased liver CASP3 in our mice, suggesting a possible positive role in preventing tumor development. Tumor necrosis factor-alpha is associated with colorectal cancers *via* inducing the expression of VEGF. In 2015, Packer and Hoffman-Goetz designed an experiment to determine the effect of a single bout of intense exercise on hippocampal expression of inflammatory mediators TNF-α and apoptotic proteases CASP3 and CASP7. These researchers used a C57BL/6 mouse model evaluating the effect of 90 min of TFR on hippocampal inflammation in young (3–4 months), middle-aged (13–14 months) and older (16–17 months) mice. The results showed post-exercise increases in hippocampal TNF-α and Caspase-3/7 in each age group (59, 60). In our study, the unexercised young mice consuming the FAS diet exhibited increased muscle TNF-α, whereas the exercised mice showed a trend of increased muscle TNF-α. Collectively, the influence of folic acid supplementation and high dietary protein intakes alongside chronic, moderate exercise of 8 weeks tended to alter key inflammatory markers that might slow cancer progression and development of dementia. Future studies will seek to measure blood, brain, liver, and microbiome crosstalk of the associated oncogenic inflammatory biomarkers that was not accomplished in this experiment.

The fecal microbiome is a principal constituent in the modulation of the metabolic response to chronic disease (26). It is rapidly becoming elucidated that connecting the dietary components and consequent metabolic degradative products of the microbiome are important feedback mechanisms to modulate the health of an organism’s metabolism (27). This study examined the indispensable dietary components, folic acid and protein, as they are considered essential in adequate protein synthesis that is requisite to mediate neurocognition and advancement of abnormal cellular proliferation as observed in cancer. Several studies highlight that nutrition and physical activity-induced changes in the gut microbiota have possible implications for human health (61–63). Indeed, a recent novel review reported heterogenous findings from a focused search of evaluating the role of physical activity as distinguished from dietary effects on the gut microbiota composition. Interestingly, of the majority studies examined, a higher value of microbial variability was found in healthy human adults as opposed to sedentary people. Specifically, a higher variability and abundance of Firmicutes was measured after physical activity. Moreover, another study examined the composition of microbiota of professional rugby players after high protein diets and prolonged exercise regimes and recorded that the athletes had a higher diversity of gut microbiota, representing 22 distinct phyla which positively correlated with protein consumption (62). A study of sedentary and exercising 6-weeks old C57BL/6NTac male mice were fed a normal or high-fat diet for 12-weeks, then duodenum/ileum bacterial communities were assayed in fecal samples. Both lean (LX) and obese exercise (OX) mice duodenum and ileum were histologically normal and members of the Clostridiales order were predominant in all diet groups whereas specific phylotypes were observed with exercise, including Faecalibacterium prausnitzi, Clostridium spp., and Allobaculum spp. (63). Some studies link diet and exercise to positive outcomes in CRC prevention, in particular examining the role of the gut microbiota in cancer-genesis as well as cancer prevention (64, 65). Several reviews of the gut microbiota of athletes discussed the concept of “health-associated” gut microbiota and have documented that exercise promoted increased microbial diversity, stimulation of bacterial abundance that can modulate mucosal immunity, and a higher abundance of health-promoting species (66–69). Gentile and Weir reinforced the significance of integrating of our dietary constituents influencing our gut microbiota and consequently, modulating our health, including specific roles of different proteins and some micronutrients (70). In this study we noted that mice consuming high dietary protein by week one, exhibited the most distinct fecal microbiota, although by week 8, both low and high dietary protein were more similar in fecal microbiota composition, and the NPC, FAD, and FAS mice had more similar fecal microbiota in week 8 too. From week 1 to week 8, there was a general shift in ratio of Firmicutes and Bacteroides, whereas, at phylum level, FAS and HPD favor an outgrowth of Verrucomicrobia by week 8. By week 8, the HPD and FAS resulted in reduction of Clostridiales_vandinBB60_group relative to the control group.

## Conclusion

Excess folic acid and high protein diets provided to C57BL/6J mice over 8 weeks induced inflammatory biomarkers commonly used to evaluate progress of chronic diseases. Surfeit folic acid and protein and exercise decreased liver IL-6 and NF kβp65, and increased liver CASP3 and muscle TNF-α. In addition, after just a week of consuming high dietary protein, the mice had distinct fecal microbiota, and by week 8, more Verrucomicrobia and less Clostridiales_vandinBB60_group, and a general shift in ratio of Firmicutes and Bacteroides. Together, these results suggested that varying dietary intake of indispensable nutrients (folic acid and protein) and chronic, moderate physical activity profoundly altered the fecal microbiota of C57BL/6J mice, both considered key modulators of our metabolic defense requisite to slow cancer progression and declining neurocognition.

## Data availability statement

The datasets presented in this study can be found in online repositories. The names of the repository/repositories and accession number(s) can be found in the article/Supplementary material.

## Ethics statement

This animal study was reviewed and approved by IUCAC–Pepperdine University.

## Author contributions

RT, SMH, and SEH designed the study. RT designed and SMH helped to implement VWR and collected Nestlet data and fecal pellets. SMH analyzed nestlets, body weights, VWR, and inflammatory biomarker data. CJ analyzed Open Field and Novel Object Recognition data. RT, SMH, CJ, GK, CG, and SEH conducted the research. CG and DG analyzed the concentration of the plasma homocysteine (HCys). LS performed the bioinformatics of the fecal microbiota and statistical analyses, interpreted the results, and addressed co-authors comments and concerns. SEH supervised all analyses, interpreted results, and mentored manuscript writing and has primary responsibility for final content. All authors read and approved the final manuscript.
