# Supplementary material for: Surfeit folic acid, protein, and exercise modify oncogenic inflammatory biomarkers and fecal microbiota
Source: Front Nutr. 2023 Jan 19;9:1060212. doi: 10.3389/fnut.2022.1060212 (PMC9894611; doi:10.3389/fnut.2022.1060212)
Supplement: Supplementary file 1 [file Data_Sheet_1.pdf]

```
data <- readRDS("susan.RDS")
##phyloseq-class experiment-level object
##otu_table()   OTU Table:         [ 919 taxa and 120 samples ]
##sample_data() Sample Data:       [ 120 samples by 6 sample variables ]
##tax_table()   Taxonomy Table:    [ 919 taxa by 7 taxonomic ranks ]

#Keep only Bacterial ASVs
ps.onlybacteria <- subset_taxa(data, Kingdom == 'Bacteria')
##phyloseq-class experiment-level object
##otu_table()   OTU Table:         [ 913 taxa and 120 samples ]
##sample_data() Sample Data:       [ 120 samples by 6 sample variables ]
##tax_table()   Taxonomy Table:    [ 913 taxa by 7 taxonomic ranks ]
##phy_tree()    Phylogenetic Tree: [ 913 tips and 912 internal node
#Initial Sum of Reads after filtering out only bacteria
sum(ps.onlybacteria@otu_table)
#5545007
#Exclude samples with less than 10,000 reads --> none excluded since all above 10,000 -- didn't run with data
we are working with
sort(phyloseq::sample_sums(ps.onlybacteria))
analysisdata <- subset_samples(ps.onlybacteria, sample_sums(ps.onlybacteria) > 10000)
#rarefy to even sequence depth - lowest sample read count is 15578
analysisdatararefy = rarefy_even_depth(analysisdata, rngseed=1, sample.size = 15578, replace = FALSE,
verbose = TRUE)

#Create Tree File with APE
random_tree = rtree(ntaxa(analysisdatararefy), rooted=TRUE, tip.label=taxa_names(analysisdatararefy))
physeq1 = merge_phyloseq(analysisdatararefy, random_tree)
physeq1
#phyloseq-class experiment-level object
#otu_table()   OTU Table:         [ 828 taxa and 120 samples ]
#sample_data() Sample Data:       [ 120 samples by 6 sample variables ]
#tax_table()   Taxonomy Table:    [ 828 taxa by 7 taxonomic ranks ]
#phy_tree()    Phylogenetic Tree: [ 828 tips and 827 internal nodes ]

#subset out weeks
week1 <- subset_samples(physeq1, Timepoint %in% c("week1"))
week5 <- subset_samples(physeq1, Timepoint %in% c("week5"))
week8 <- subset_samples(physeq1, Timepoint %in% c("week8"))

#week 1/5/8 richness - switch out for different subsets
richnesschao1 <- plot_richness(week1, "Diet", measures=c("Observed", "Chao1", "Shannon")) +
  +     geom_boxplot() +
  +     theme_classic() +
  +     xlab("") + ylab("") +
  +     ggtitle("") +
  +     theme(axis.title.x=element_text(size=20)) +
  +     theme(axis.title.y=element_text(size=20)) +
```

```
  +     theme(axis.text.x=element_text(size=20)) +
  +     theme(axis.text.y=element_text(size=20)) +
  +     theme(legend.text=element_text(size=20)) +
  +     theme(legend.title=element_text(size=20)) + scale_x_discrete(labels = c("FPC", "NFA", "FAS", "LPD",
"HPD"))
richnesschao1

#Shapiro-Wilk test for normality for week 1 - repeat for weeks 5 and 8
results <- estimate_richness(week1, measures = 'Chao1')
d = sample_data(week1)
res <- cbind(results, d)
resultsS <- estimate_richness(week1, measures = 'Shannon')
resS <- cbind(resultsS, res)
resultsO <- estimate_richness(week1, measures = 'Observed')
resO <- cbind(resultsO, res)
shapiro.test(resO$Shannon)

#all returned p > 0.05 weeks 1 and 5 and 8

#One-way ANOVA for week 1 - repeat for weeks 5 and 8
aov<- aov(Shannon~Diet, data=resS)
summary(aov)
#Df Sum Sq Mean Sq F value   Pr(>F)
#Diet         4  1.500  0.3749   11.77 3.67e-06 ***
  #Residuals   35  1.115  0.0319

  #Signif. codes:  0 ‘***’ 0.001 ‘**’ 0.01 ‘*’ 0.05 ‘.’ 0.1 ‘ ’ 1
TukeyHSD(aov)

#Tukey multiple comparisons of means - week 1
#95% family-wise confidence level

#Fit: aov(formula = Shannon ~ Diet, data = resS)

#$Diet
#diff         lwr         upr     p adj
#FPC-FAS  0.18552339 -0.07105143  0.44209821 0.2517778
#HPD-FAS  0.21280189 -0.04377293  0.46937671 0.1435291
#LPD-FAS -0.32644516 -0.58301998 -0.06987034 0.0069521
#NFA-FAS -0.03781109 -0.29438591  0.21876373 0.9929629
#HPD-FPC  0.02727850 -0.22929632  0.28385332 0.9980078
#LPD-FPC -0.51196855 -0.76854337 -0.25539373 0.0000163
#NFA-FPC -0.22333448 -0.47990929  0.03324034 0.1131499
#LPD-HPD -0.53924705 -0.79582187 -0.28267223 0.0000065
#NFA-HPD -0.25061298 -0.50718780  0.00596184 0.0582837
#NFA-LPD  0.28863407  0.03205925  0.54520889 0.0209781
```

```
#nothing significant for observed, nothing for Chao1 either

#Tukey comparison of means week 5 - shannon
#$Diet
#diff         lwr         upr     p adj
#FPC-FAS -0.31745888 -0.53827436 -0.09664339 0.0018645
#HPD-FAS  0.23694307  0.01612758  0.45775855 0.0303525
#LPD-FAS -0.42018091 -0.64099640 -0.19936543 0.0000363
#NFA-FAS  0.18181432 -0.03900117  0.40262980 0.1484840
#HPD-FPC  0.55440194  0.33358646  0.77521743 0.0000002
#LPD-FPC -0.10272204 -0.32353752  0.11809345 0.6702458
#NFA-FPC  0.49927320  0.27845771  0.72008868 0.0000016
#LPD-HPD -0.65712398 -0.87793947 -0.43630849 0.0000000
#NFA-HPD -0.05512875 -0.27594423  0.16568674 0.9509942
#NFA-LPD  0.60199523  0.38117975  0.82281072 0.0000000

#$Diet - week 5 observed
#diff        lwr       upr     p adj
#FPC-FAS   0.375 -19.705817 20.455817 0.9999980
#HPD-FAS  18.875  -1.205817 38.955817 0.0738707
#LPD-FAS  -8.125 -28.205817 11.955817 0.7717345
#NFA-FAS   8.000 -12.080817 28.080817 0.7814496
#HPD-FPC  18.500  -1.580817 38.580817 0.0830688
#LPD-FPC  -8.500 -28.580817 11.580817 0.7416730
#NFA-FPC   7.625 -12.455817 27.705817 0.8095651
#LPD-HPD -27.000 -47.080817 -6.919183 0.0039451
#NFA-HPD -10.875 -30.955817  9.205817 0.5338327
#NFA-LPD  16.125  -3.955817 36.205817 0.1662572

#$Diet - week 5 chao1
#diff        lwr      upr     p adj
#FPC-FAS   0.5562771 -20.613921 21.72648 0.9999922
#HPD-FAS  19.8171131  -1.353085 40.98731 0.0756998
#LPD-FAS  -7.8825758 -29.052774 13.28762 0.8202074
#NFA-FAS   8.6735119 -12.496687 29.84371 0.7636744
#HPD-FPC  19.2608360  -1.909362 40.43103 0.0891876
#LPD-FPC  -8.4388528 -29.609051 12.73135 0.7810947
#NFA-FPC   8.1172348 -13.052964 29.28743 0.8041060
#LPD-HPD -27.6996889 -48.869887 -6.52949 0.0052471
#NFA-HPD -11.1436012 -32.313800 10.02660 0.5609864
#NFA-LPD  16.5560877  -4.614111 37.72629 0.1862052


#$Diet - week 8 shannon - nothing less than 0.05
#diff        lwr        upr     p adj
#FPC-FAS  0.11085209 -0.1855713 0.40727547 0.8178888
##HPD-FAS -0.11299405 -0.4094174 0.18342933 0.8074026
```

```
#LPD-FAS  0.04654184 -0.2498815 0.34296521 0.9910436
#NFA-FAS -0.13809896 -0.4345223 0.15832442 0.6690348
#HPD-FPC -0.22384614 -0.5202695 0.07257724 0.2143534
#LPD-FPC -0.06431025 -0.3607336 0.23211312 0.9702557
#NFA-FPC -0.24895105 -0.5453744 0.04747233 0.1352344
#LPD-HPD  0.15953589 -0.1368875 0.45595927 0.5398252
#NFA-HPD -0.02510491 -0.3215283 0.27131847 0.9991835
#NFA-LPD -0.18464080 -0.4810642 0.11178258 0.3948093


#$Diet - week 8 observed
#diff        lwr       upr     p adj
#FPC-FAS  -3.000 -30.117823 24.117823 0.9976738
#HPD-FAS -31.750 -58.867823 -4.632177 0.0149998
#LPD-FAS  -8.750 -35.867823 18.367823 0.8842184
#NFA-FAS -22.125 -49.242823  4.992823 0.1548395
#HPD-FPC -28.750 -55.867823 -1.632177 0.0331982
#LPD-FPC  -5.750 -32.867823 21.367823 0.9726247
#NFA-FPC -19.125 -46.242823  7.992823 0.2743836
#LPD-HPD  23.000  -4.117823 50.117823 0.1289251
#NFA-HPD   9.625 -17.492823 36.742823 0.8442220
#NFA-LPD -13.375 -40.492823 13.742823 0.6205190


#$Diet - week 8 chao1
#diff        lwr       upr     p adj
#FPC-FAS  -2.577173 -33.488913 28.334568 0.9992325
#HPD-FAS -36.608007 -67.519747 -5.696266 0.0135732
#LPD-FAS -10.554995 -41.466736 20.356746 0.8616669
#NFA-FAS -25.429413 -56.341154  5.482328 0.1490948
#HPD-FPC -34.030834 -64.942575 -3.119093 0.0249285
#LPD-FPC  -7.977822 -38.889563 22.933919 0.9449985
#NFA-FPC -22.852240 -53.763981  8.059500 0.2323567
#LPD-HPD  26.053012  -4.858729 56.964752 0.1329494
#NFA-HPD  11.178593 -19.733147 42.090334 0.8351765
#NFA-LPD -14.874418 -45.786159 16.037322 0.6419823

#Beta Diversity - week 1/5/8 -- change out subset
ord1 <- ordinate(week1, "PCoA", "wuniFrac") #change to "unifrac" for unweighted
plot_ord1 <- plot_ordination(week1, ord1, color="Diet",title="Week 1") +
  +     stat_ellipse(level = 0.95) + geom_point(size=4) +
  +     theme_bw() +
  +     scale_color_manual(values=c("red","black","purple", "orange", "blue"), breaks = c("FAS", "NFA", "FPC",
"LPD", "HPD")) +
  +     theme(axis.title.x=element_text(size=20)) +
  +     theme(axis.title.y=element_text(size=20)) +
  +     theme(axis.text.x=element_text(size=20)) +
```

```
+     theme(axis.text.y=element_text(size=20)) +
+     theme(legend.text=element_text(size=20)) +
+     theme(legend.title=element_text(size=20))

#BetaDisper -- checking for homogeneity/distance from centroid
distance = distance(week1, "UniFrac") #weighted, add weighted = F for unweighted
groups <- sample_data(week1)$Diet
mod <- betadisper(distance, groups)
permutest(mod)

#p 0.178 for week 1, p 0.036 for week 5 (can't apply permanova), 0.088 for week 8
#p unweighted week 1 is 0.183, p for week five = 0.038 (can't apply permanova), p for week 8 is 0.095.
mod.HSD <- TukeyHSD(mod)
plot(mod.HSD)

#permanova
dist = distance(week1, "unifrac")
adonis(dist ~ Diet, as(sample_data(week1), "data.frame"))
#week 1 p = 0.001 for both weighted and unweighted, same for week 8, just means sample clustering is
significant

#Relative abundance

pruned <- prune_taxa(taxa_sums(analysisdata)>=20, analysisdata) #remove ASVs with less than 20 reads
week1_pruned <- subset_samples(pruned, Timepoint %in% c("week1")) #subset by timepoint
week5_pruned <- subset_samples(pruned, Timepoint %in% c("week5"))
week8_pruned <- subset_samples(pruned, Timepoint %in% c("week8"))
merge_1 = merge_samples(week1_pruned, "Diet") #categorical merge per week
sample_data(merge_1)$Diet<- levels(sample_data(week1_pruned)$Diet)
merge_5 = merge_samples(week5_pruned, "Diet")
sample_data(merge_5)$Diet<- levels(sample_data(week5_pruned)$Diet)
merge_8 = merge_samples(week8_pruned, "Diet")
sample_data(merge_8)$Diet<- levels(sample_data(week8_pruned)$Diet)
merge.100_1 = transform_sample_counts(merge_1, function(x) 100 * x/sum(x)) #transform to relative
abundance per week
merge.100_5 = transform_sample_counts(merge_5, function(x) 100 * x/sum(x))
merge.100_8 = transform_sample_counts(merge_8, function(x) 100 * x/sum(x))

#week 1
sample_data(merge.100_1)$Diet <- factor(sample_data(merge.100_1)$Diet, levels = c("FPC", "NFA", "FAS",
"LPD", "HPD"))
levels(sample_data(merge.100_1)$Diet) #relevel

p <- plot_bar(merge.100_1, "Diet", "Abundance", "Phylum") +
  xlab("Classification") +
  ylab("Abundance (%)") +
  ggtitle("Relative Abundance Plot") +
```

```
  geom_bar(aes(color=Phylum, fill=Phylum),
           stat="identity", position='stack') +
  theme_classic() +
  theme(axis.title.x=element_text(size=20)) +
  theme(axis.title.y=element_text(size=20)) +
  theme(axis.text.x=element_text(size=20)) +
  theme(axis.text.y=element_text(size=20)) +
  theme(legend.text=element_text(size=20)) +
  theme(legend.title=element_text(size=20)) +scale_x_discrete(labels = c("Control (FPC)", "Folic Acid Deficient (NFA)", "Folic Acid Supplemented (FAS)", "Low Protein Diet (LPD)", "High Protein Diet (HPD)")) #change phylum to family for family plot, change to week 5 and 8 for different weeks

#Maaslin
write.csv(week1_pruned@tax_table, 'week1_taxadesignation6.csv')
week1_maaslin <- read.csv('week1_taxadesignation6.csv') #add in title ASV to ASV column and create a new column with a unique number and taxa id
rownames(week1_pruned@tax_table) <- as.factor(week1_maaslin$ASV)
colnames(week1_pruned@otu_table) <- as.factor(week1_maaslin$ASV)
analysisdata_pruned_abund <- microbiome::transform(week1_pruned,
                                          transform = "compositional",
                                          target = "OTU", shift = 0,
                                          scale = 1)

input_data <- as.data.frame(analysisdata_pruned_abund@otu_table)
input_data <- as.data.frame(t(input_data))
rownames(input_data) <- as.factor(week1_maaslin$ID)#replace ASV nucleotides with ID
meta <- as.matrix(analysisdata_pruned_abund@sam_data)
meta <- as.data.frame(meta)
fit_data = Maaslin2(input_data = input_data, input_metadata = meta, output = "maaslin2_output_Susan", fixed_effects = c("Diet"),reference = c("Diet,FPC"), plot_heatmap = TRUE)

#Plot top 50 significant associations
fit_data_df <- as.data.frame(fit_data$results)
fit_data_df_sig <- subset(fit_data_df, qval < 0.006)

maaslin2 <- ggplot(fit_data_df_sig, aes(x=coef, y=feature, color = value, shape = value)) +
  theme_classic() +xlab("Coefficient") +
  geom_errorbar(aes(xmin=coef-stderr,xmax=coef+stderr), width=.2,position=position_dodge(0.25)) +
 geom_point(size=3, position=position_dodge(0.25),aes(fill=value, color=value))+
ylab("") + ggtitle("")+
xlab("") + xlab("Coefficient") + ylab("Feature") + scale_fill_manual(values=c('black', 'green', 'orange', 'purple'))+
scale_color_manual(values=c('black', 'red', 'orange', 'blue')) +
scale_shape_manual(values=seq(0,10))+
theme(axis.title.x=element_text(size=10)) +
theme(axis.title.y=element_text(size=10)) +
theme(axis.text.x=element_text(size=10)) +
theme(axis.text.y=element_text(size=8)) +theme(legend.text=element_text(size=10)) +
```

```
theme(legend.title=element_text(size=10))
maaslin2

#Maaslin week 5
write.csv(week8_pruned@tax_table, 'week8_taxadesignation3.csv')
week8_maaslin <- read.csv('week8_taxadesignation1.csv') #add in title ASV to ASV column and create a new
column with a unique number and taxa id
rownames(week8_pruned@tax_table) <- as.factor(week1_maaslin$ASV) #taxa table is the same so used
week 1
colnames(week8_pruned@otu_table) <- as.factor(week1_maaslin$ASV)
analysisdata_pruned_abund <- microbiome::transform(week8_pruned,
                                         transform = "compositional",
                                         target = "OTU", shift = 0,
                                         scale = 1)

input_data <- as.data.frame(analysisdata_pruned_abund@otu_table)
input_data <- as.data.frame(t(input_data))
rownames(input_data) <- as.factor(week1_maaslin$ID)#replace ASV nucleotides with ID
meta <- as.matrix(analysisdata_pruned_abund@sam_data)
meta <- as.data.frame(meta)
fit_data = Maaslin2(input_data = input_data, input_metadata = meta, output =
"maaslin2_output_Susanweek8", fixed_effects = c("Diet"),reference = c("Diet,FPC"), plot_heatmap = TRUE)

#Plot top 50 significant associations
fit_data_df <- as.data.frame(fit_data$results)
fit_data_df_sig2 <- subset(fit_data_df, qval < 2.53e-02)

maaslin2 <- ggplot(fit_data_df_sig2, aes(x=coef, y=feature, color = value, shape = value)) +
  theme_classic() +xlab("Coefficient") +
  geom_errorbar(aes(xmin=coef-stderr,xmax=coef+stderr), width=.2,position=position_dodge(0.25)) +
  geom_point(size=3, position=position_dodge(0.25),aes(fill=value, color=value))+
  ylab("") + ggtitle("")+
  xlab("") + xlab("Coefficient") + ylab("Feature") + scale_fill_manual(values=c('black', 'green', 'orange',
'purple'))+ scale_color_manual(values=c('black', 'red', 'orange', 'blue')) +
  scale_shape_manual(values=seq(0,10))+
  theme(axis.title.x=element_text(size=10)) +
  theme(axis.title.y=element_text(size=10)) +
  theme(axis.text.x=element_text(size=10)) +
  theme(axis.text.y=element_text(size=8)) +theme(legend.text=element_text(size=10)) +
  theme(legend.title=element_text(size=10))
maaslin2
```
